# COVID-19 in the United States: Trajectories and second surge behavior

**DOI:** 10.1063/5.0024204

**Published:** 2020-09-22

**Authors:** Nick James, Max Menzies

**Affiliations:** 1 School of Mathematics and Statistics, University of Sydney, NSW 2006, Australia; 2 Yau Mathematical Sciences Center, Tsinghua University, Beijing 100084, China

## Abstract

This paper introduces a mathematical framework for determining second surge behavior of COVID-19 cases in the United States. Within this framework, a flexible algorithmic approach selects a set of turning points for each state, computes distances between them, and determines whether each state is in (or over) a first or second surge. Then, appropriate distances between normalized time series are used to further analyze the relationships between case trajectories on a month-by-month basis. Our algorithm shows that 31 states are experiencing second surges, while four of the 10 largest states are still in their first surge, with case counts that have never decreased. This analysis can aid in highlighting the most and least successful state responses to COVID-19.

The United States U.S. has been severely impacted by COVID-19 and leads the world in both cases and death counts.^1^ Individual states have largely determined their own response to the pandemic,^2^ seeking to protect citizens’ lives while mitigating economic consequences. Following lockdowns and business closures in March and April, all 50 states reduced their restrictions.^3^ Since then, the U.S. has experienced a severe rise in new cases, with a public debate on whether or not to term this a “second surge.”^4,5^ A careful identification of most and least successful states is, therefore, of great relevance to a response to the ongoing threat of COVID-19. This paper has two goals: to develop a mathematical framework that defines a second surge and identifies the states that are experiencing one and to compare the trajectories of new case counts as a means of determining effective pandemic responses.

## I. INTRODUCTION

Understanding the trajectory of COVID-19 case counts assists governments in responding to the impact of the pandemic. Given the highly infectious nature of the disease, an increasing number of new daily cases may overwhelm the healthcare system and prompt further restrictions on businesses and social gatherings. Conversely, a decreasing number of new cases is a good sign but should be carefully monitored. Thus, the *turning points* in the new case counts are crucial to identify. Given the randomness of human behavior, however, turning points are difficult to predict and predictive modeling of COVID-19 requires frequent observation of real-time data. This paper focuses on a retrospective analysis of case trajectories in different U.S. states to determine which public policy responses were most and least effective. While we focus on the U.S., our methodology may be applied more broadly to understand the impact of public policies on countries with similar federative structures such as Brazil and India. These three countries have the highest COVID-19 case counts in the world,^1^ with government responses differing between states and yielding differing results.^6,7^

For this goal, we use existing and new techniques from *time series analysis*. Time series analysis has been widely applied to epidemiology,^8,9^ including COVID-19.^10–12^ Existing methods of time series analysis are diverse, including power-law models^13^ and nonparametric methods such as distance analysis,^14^ distance correlation,^15–17^ and network models.^18^ In this paper, we aim to develop a new mathematical framework of identification and comparison of *turning points* of time series to study the spread of COVID-19 in the U.S. Such turning points classify the behavior of states’ trajectories throughout the pandemic as being in (or over) their first surge or second surge.

In addition to the aforementioned state-by-state determination of turning points, this paper uses a new application of semi-metrics to measure distance between the states’ behaviors and performs clustering based on this. The paper implements *hierarchical clustering*,^19,20^ which has previously been used in various epidemiological applications. These include inflammatory diseases,^21^ airborne diseases,^22^ Alzheimer’s disease,^23^ Ebola,^24^ SARS,^25^ and COVID-19.^11^

The paper is structured as follows: in each of Secs. II and III, we introduce portions of our methodology and then present our results. Section II describes our framework for identifying turning points, determining which states are in a second surge, and clustering based on similar behavior. Section III analyzes the trajectories of COVID-19 counts in each state on a month-by-month basis. Section IV summarizes the results and new findings regarding the spread of COVID-19 in the United States. In Appendix A, we briefly apply our analysis to the Brazilian states to demonstrate the generality of our method.

## II. SECOND SURGE ANALYSIS

In this section, we develop a mathematical framework and procedure to determine whether a state has experienced a second surge. Through the careful selection of turning points, we formulate a definition applicable to an individual time series and develop a method for comparing differing surge behaviors among a collection of time series.

### A. Second surge methodology: Determination of turning points

Let $x_{i}(t)\in R$ be a collection of real-valued time series over a common time interval t=1,…,T,i=1,…,n. In this paper, the analyzed time series are the daily counts of new cases in the 50 U.S. states and the District of Columbia (D.C.), ordered alphabetically, i=1,…,51. Our data span from 01/21/2020 to 07/31/2020, a period of T=193 days across n=51 regions.

There are several irregular features in the dataset, including lower case counts on the weekends, some negative daily counts due to adjustments of previous figures, and general noise. In addition, there are small disparities between different data sources. In order to isolate the signal in a dataset and between different datasets, we first apply a *Savitzky–Golay filter*^26^ to the counts to produce a collection of *smoothed time series* ${\hat{x}}_{i}(t)$, t=1,…,T,i=1,…,n. This combines a moving average calculation with polynomial smoothing. Through its moving average computations, it largely eliminates all negative counts, except when there are very few cases. In these instances, we replace any negative smoothed count with a zero. For the remainder of the paper, we analyze these smoothed cases ${\hat{x}}_{i}(t)$. Due to the smoothing process, ${\hat{x}}_{i}(t)\in R_{\geq 0}$ are not necessarily integers but are all non-negative.

Our identification of turning points and second surge behavior of a smoothed time series $\hat{x}(t)$ proceeds in two steps. First, we identify a sequence of potential local maxima or *peaks*, and local minima or *troughs*. Second, we appropriately refine this sequence according to chosen conditions. The final sets P and T of peaks and troughs, respectively, determine whether the time series is said to be in (or over) its first or second surge. It will be essential that P and T are non-empty.

**FIG. 1.**
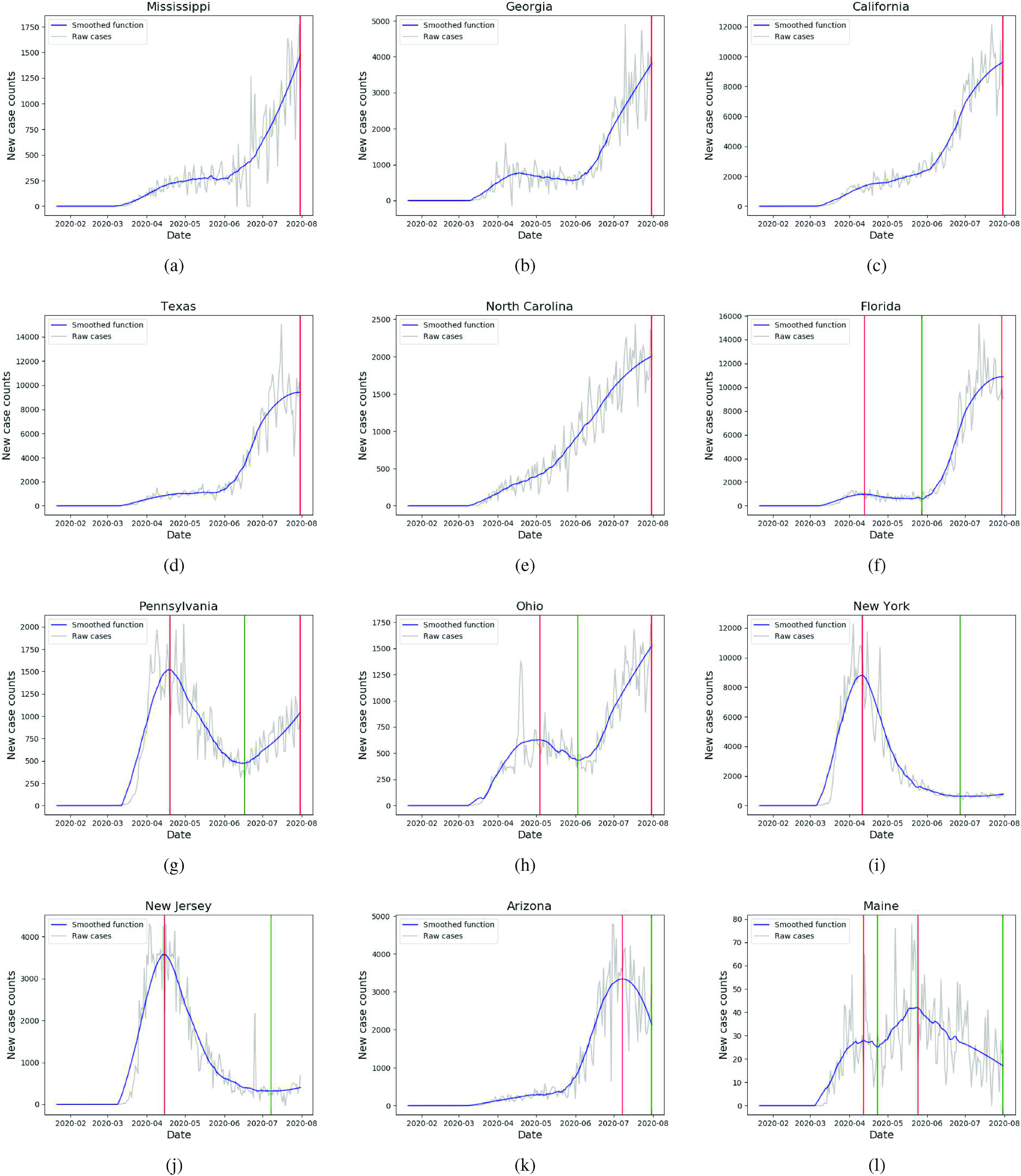
Smoothed time series and identified turning points for various states: (a) Mississippi, (b) Georgia, (c) California, (d) Texas, and (e) North Carolina are assigned sequence TP and determined to be in their first surge. (f) Florida, (g) Pennsylvania, and (h) Ohio are determined to be in their second surges, with sequence TPTP. (i) New York and (j) New Jersey are assigned sequence TPT and determined to have concluded their first surge and flattened the curve. (k) Arizona and (l) Maine are assigned TPT and TPTPT with final trough at the end of the period and determined to be declining from their first and second surges, respectively.

For the first step, the basic idea is to designate a time $t_{0}$ a peak or trough, respectively, if

$$\begin{array}{rl} \hat{x}(t_{0}) & =\max\{\hat{x}(t):\max(1,t_{0}-l)\leq t\leq \min(t_{0}+l,T)\}, \end{array}$$ (1)

$$\begin{array}{rl} \hat{x}(t_{0}) & =\min\{\hat{x}(t):\max(1,t_{0}-l)\leq t\leq \min(t_{0}+l,T)\}, \end{array}$$ (2)

for a parameter $l<\frac{T}{2}$, the length over which we look. In this paper, we select l=17 to account for the 14-day incubation period of the virus^27^ and reduced testing on weekends. These naïve definitions of (1) and (2) have two flaws: first, equal values of $\hat{x}(t)$ may determine consecutive values of t as peaks or troughs when only one should be counted. More subtly, it is possible that two troughs may be detected at two points that are far apart, with no peak between them, when the time series has been largely monotonic between the two. For example, in Fig. 1(a), troughs are naïvely detected at $t_{0}=1$ and 126, corresponding to the start of the time series and 05/26/2020, respectively. What follows is a method to exclude the latter.

We implement variants of (1) and (2) by sequentially examining the values of $\hat{x}(t)$. The first peak or trough is assigned at the first value of $t_{0}$ such that (1) or (2) holds, respectively. For each U.S. state, this is an initial trough at $t_{0}=1$ corresponding to zero cases there after smoothing. Having determined a peak at $t_{0}$, we search in the period $t>t_{0}$ for one of two elements: if we find a trough at $t_{1}>t_{0}$ according to (2), we add it to the set of troughs and proceed from $t_{1}$ as normal. If we find a peak at $t_{1}>t_{0}$ according to (1) such that $\hat{x}(t_{0})\geq \hat{x}(t_{1})$, we ignore this lesser peak as redundant; if we find a peak at $t_{1}>t_{0}$ according to (1) such that $\hat{x}(t_{0})<\hat{x}(t_{1})$, we remove peak $t_{0}$ and replace it with $t_{1}$ and continue from there. An analogous process applies from a trough at $t_{0}$. With this process, we generate an alternating sequence of troughs and peaks, starting with a trough at $t_{0}=1$. Every time series is assigned at least one peak and trough at its global maximum and minimum, respectively. If the global maximum is not unique, a peak is assigned at the first maximum. This concludes the first step.

So far, every time series in our collection is assigned an alternating sequence of peaks and troughs beginning with a trough at $t_{0}=1$. One could naïvely define a state as being in its second surge if its sequence so far is TPTP. However, some detected peaks and troughs are immaterial and should be excluded. We describe a flexible approach to excluding trivial peaks or troughs, in which we apply two conditions to do so.

Let $t_{1}<t_{3}$ be two peaks, necessarily separated by a trough. We select a parameter δ and if the *peak ratio*, defined as $\frac{\hat{x}(t_{3})}{\hat{x}(t_{1})}<\delta$, we remove the peak $t_{3}$. If two consecutive troughs $t_{2},t_{4}$ remain, we remove $t_{2}$ if $\hat{x}(t_{2})>\hat{x}(t_{4})$, otherwise remove $t_{4}$. That is, if the second peak has size less than δ of the first peak, we remove it, deciding not to term it a second surge.

Finally, we define the *log-gradient* between times $t_{1}<t_{2}$ as

$$\log-\mathrm{grad}(t_{1},t_{2})=\frac{\log\hat{x}(t_{2})-\log\hat{x}(t_{1})}{t_{2}-t_{1}}.$$ (3)

The numerator coincides with $\log(\frac{\hat{x}(t_{2})}{\hat{x}(t_{1})})$ and is a more appropriate substitution for the “rate of increase” given by $\frac{\hat{x}(t_{2})}{\hat{x}(t_{1})}-1$. Indeed, a “rate of increase” is asymmetrically bounded between (−1,∞), while the logarithmic rate is bounded between (−∞,∞). The log−grad function measures the average rate of logarithmic increase over the period $\left[t_{1},t_{2}\right]$. Now, let $t_{1},t_{2}$ be an adjacent peak and trough. We select a parameter ϵ=0.01, if

$$|\log-\mathrm{grad}(t_{1},t_{2})|<\epsilon ,$$ (4)

that is, the average logarithmic increase or decrease is well-defined and less than 1%, we remove both $t_{1}$ and $t_{2}$ from our sets of peaks and troughs. For example, this step removes a peak and trough from Fig. 1(b), where the local maximum at 04/17/2020 is immaterial. This condition always preserves the trough at $t_{0}=1$, where $\hat{x}(t_{0})=0$ and the peak at the global maximum. This concludes the selection of P and T.

To quantify distance between time series’ turning points, we apply the semi-metrics of Ref. 28 (with p=1). Given two non-empty finite sets $S_{1},S_{2}$, this is defined as

$$D(S_{1},S_{2})=\frac{1}{2}\left(\frac{\sum_{b\in S_{2}}d(b,S_{1})}{|S_{2}|}+\frac{\sum_{a\in S_{1}}d(a,S_{2})}{|S_{1}|}\right),$$ (5)

where $d(b,S_{1})$ is the minimal distance from $b\in S_{2}$ to the set $S_{1}$. The values $d(S_{1},S_{2})$ are symmetric, non-negative, and zero if and only if $S_{1}=S_{2}.$ Then, we define the n×n *surge behavior matrix* between turning point sets by

$$D_{ij}^{TP}=D(P_{i},P_{j})+D(T_{i},T_{j}).$$ (6)

Then, $D_{ij}^{TP}=0$ if and only if time series ${\hat{x}}_{i}(t)$ and ${\hat{x}}_{j}(t)$ have equal sets of peaks and troughs, hence identical surge behavior. These procedures are presented in an algorithmic format in Appendix B.

### B. Second surge analysis results

**FIG. 2.**
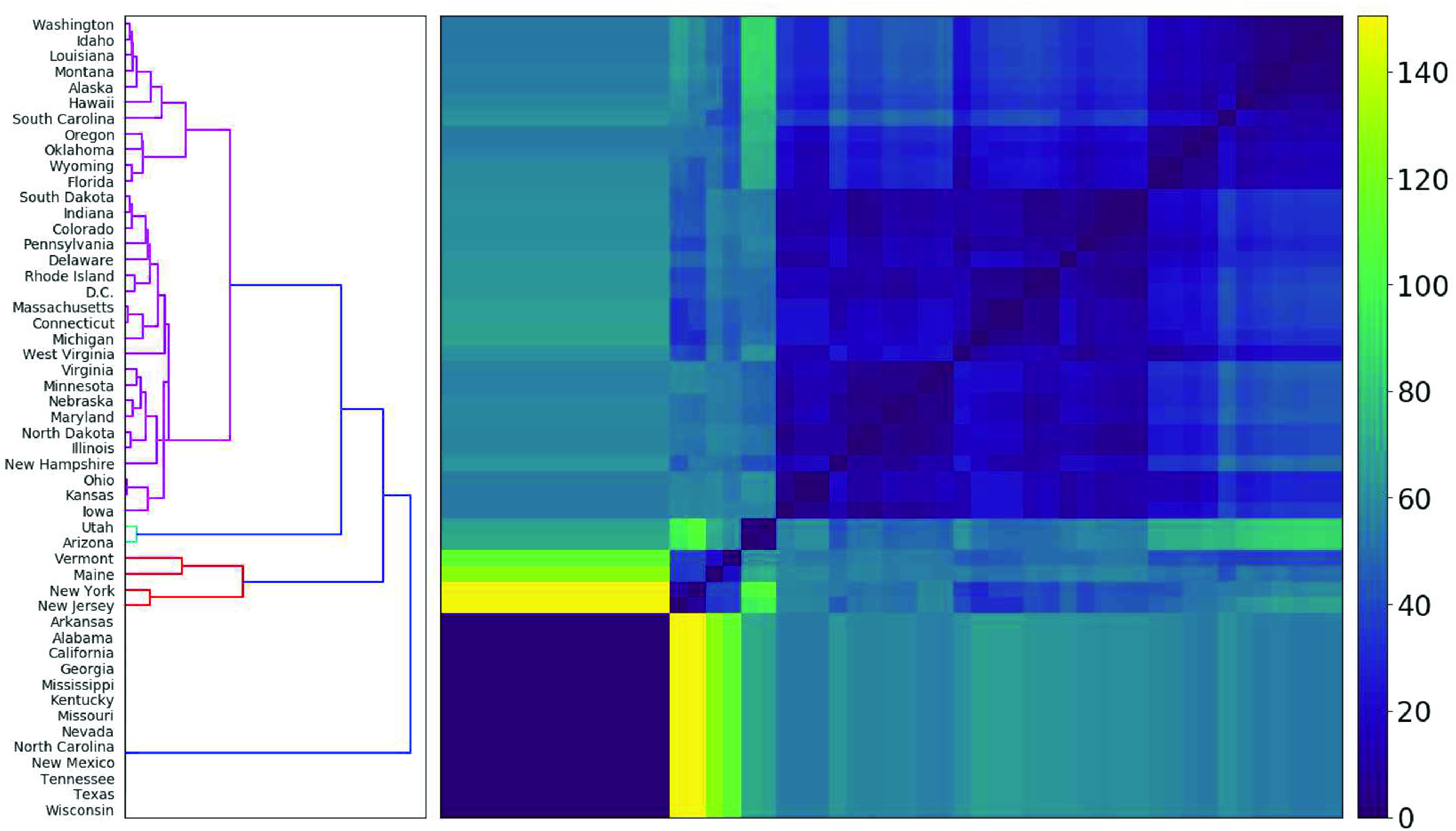
*Surge behavior matrix*, defined in Sec. II, measures distance between sets of turning points in new case trajectories. Five primary (sub)clusters of time series are identified with the following behaviors: 13 states in their first surge, two states that have completed their first surge and flattened the curve, two states coming down from their first surge, 31 states plus D.C. that are beyond their first surge and are now experiencing a second surge and two states coming down from their second surge.

Our methodology assigns one of four possible sequence types to each state. Thirteen states, including Georgia, California, Texas, and North Carolina [Figs. 1(b), 1(c), 1(d), and 1(e), respectively] are assigned the sequence TP (that is, one trough, then one peak) and deemed to be in their first surge. All 13 of these have their unique peak and global maximum on the final day and form a cluster of identical similarity in Fig. 2, where we implement hierarchical clustering on $D^{TP}$. Identical results are obtained with any value δ∈[0.1,0.2], so we select δ=0.2. That is, we exclude any second surge that is less than a fifth of the first. Then, 31 states plus D.C. are assigned TPTP—we deem these to be in their second surge. The three largest of these second surge states are Florida, Pennsylvania, and Ohio, displayed in Figs. 1(f), 1(g), and 1(h), respectively. Of these, all but Florida and South Carolina have a peak on the final day, with 19 exhibiting their global max on that day. These second surge states form the majority cluster in Fig. 2. Four states are assigned the sequence TPT of which New York and New Jersey [1(i) and 1(j)] have a local max removed due to a peak ratio of less than 0.2. Their curves have been flattened, and the first surge is completely over. Arizona [1(k)] and Utah are also assigned TPT, with their latter trough on the final day, indicating they are still coming down from a first surge. Finally, Maine [1(l)] and Vermont are assigned the sequence TPTPT. For Maine, this final trough is at the end of the period, indicating it is still coming down from its second surge, while Vermont’s final trough is before the end, indicating it has flattened the curve on its second surge. Hierarchical clustering on $D^{TP}$ distinguishes all these behaviors into separate clusters.

## III. DYNAMIC TRAJECTORY MODELING

In this section, we further analyze the new case counts in the 50 states and D.C., examining the trajectories of smoothed case counts on a month-by-month basis. Restricting these smoothed counts to a particular month gives a sequence $f_{i}=(f_{i}(1),f_{i}(2),\ldots ,f_{i}(m)))\in R^{m}$, where m∈{29,30,31} is the number of days in that month and i=1,…,51.

Let $||f_{i}||=\sum_{t=1}^{m}|f_{i}(t)|$ be the $L^{1}$ norm of the vector $f_{i}$. As all $f_{i}(t)$ are non-negative, this counts the total number of new cases (up to smoothing) observed in a month and is non-zero for every state and month after March. Thus, we may define $g_{i}=\frac{f_{i}}{||f_{i}||}$. The vectors $g_{i}$ reflect relative changes of new case counts within a month. For example, a state whose new cases in a month differ between 1000 and 1100 will present a relatively flat normalized trajectory; whereas a state whose new cases in a month rise from 0 to 100 will present a more steeply increasing normalized trajectory, as a reflection of the relative change. We define *trajectory distance matrices* $D_{ij}=||g_{i}-g_{j}||$ that measure distance between normalized trajectories. This distance differs from the frequently used *distance correlation*,^10,15–17^ which is a more suitable measure between *cumulative cases*. First, distance correlation is equal to 1 when two sequences have the greatest possible similarity, whereas the distance $D_{ij}$ between two identical sequences is 0. Secondly, whereas sequences (1,2,3,4) and (4,3,2,1) have distance correlation equal to 1, they have significantly different normalized trajectory distance, heralding the fact that one sequence is increasing while the other is decreasing. Specifically, $||g_{i}-g_{j}||=0$ if and only if $f_{i}=\alpha f_{j}$ for some α>0, while two sequences $\left(X_{k}),(Y_{k}\right)$ have distance correlation 1 if and only if $X_{k}=a+bY_{k}$ for constants a,b. That is, distance correlation does not distinguish between positive and negative gradients.

**FIG. 3.**
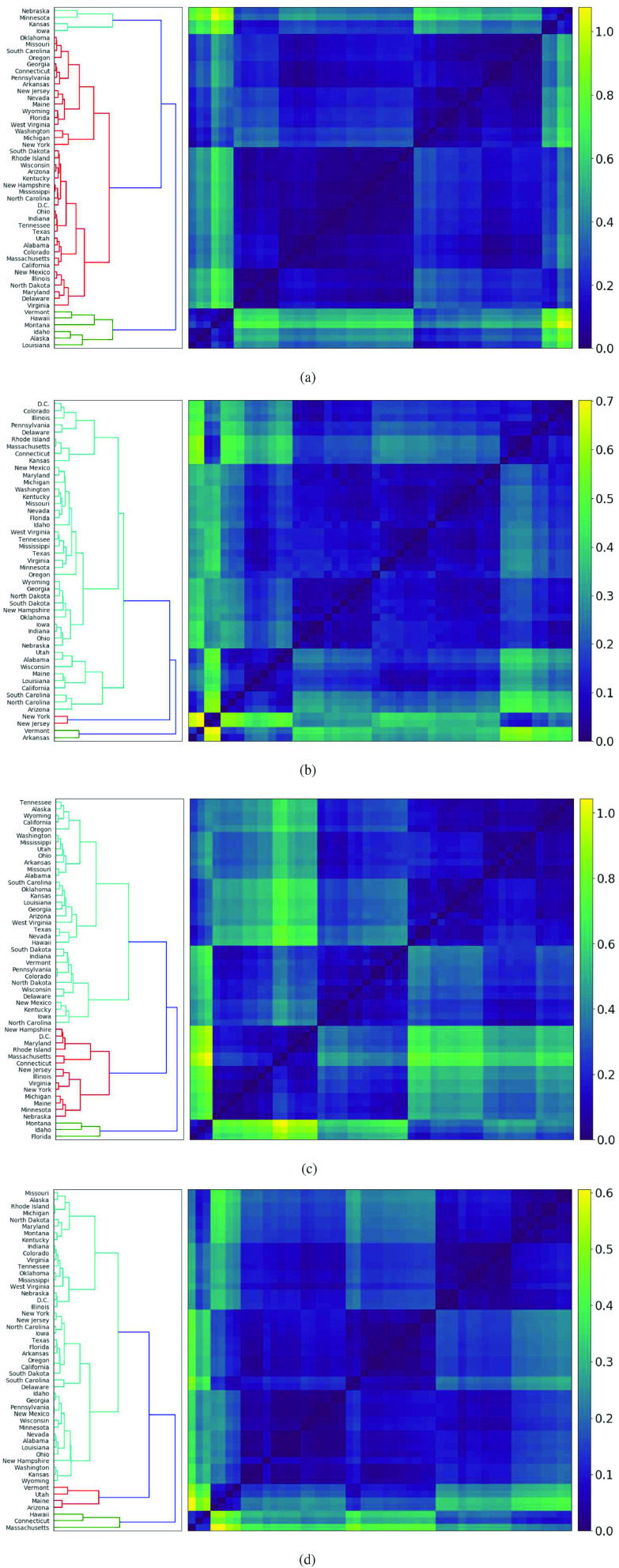
*Trajectory dendrograms*, defined in Sec. III for the months of (a) April, (b) May, (c) June, and (d) July. These dendrograms highlight states with similar new case trajectories on a month-by-month basis. For all four months, there is a consistent cluster structure: two small clusters and a large cluster with several concentrated subclusters.

In Figs. 3(a)–3(d), respectively, we implement hierarchical clustering on these matrices for the months of April, May, June, and July. There is consistent similarity in the dendrogram structure: every figure has three clusters, two small clusters, and one majority cluster that contains several subclusters of high internal similarity. The two small clusters generally consist of states that are experiencing steeply increasing or decreasing trajectories, while the larger cluster exhibits more heterogeneity. We describe the common features of the dendrograms in Table I. There, we also include the *Frobenius norm* of each distance matrix. For an n×n matrix A, this is defined as $||A||={\left(\sum_{i,j=1}^{n}|a_{ij}{|}^{2}\right)}^{\frac{1}{2}}$ and quantifies the total spread of all distances in a month.

**TABLE I.** Number of clusters, cluster sizes, and Frobenius norm for trajectory distance matrices over four months.

Trajectory distance matrices			
Month	Clusters	Cluster sizes	Frobenius norm
April	3	{4,6,41}	15.34
May${}^{a}$	3	{2,2,44}	15.97
June	3	{3,14,34}	18.35
July	3	{3,4,44}	8.67

${}^{a}$Three states are excluded in May due to low counts.

**FIG. 4.**
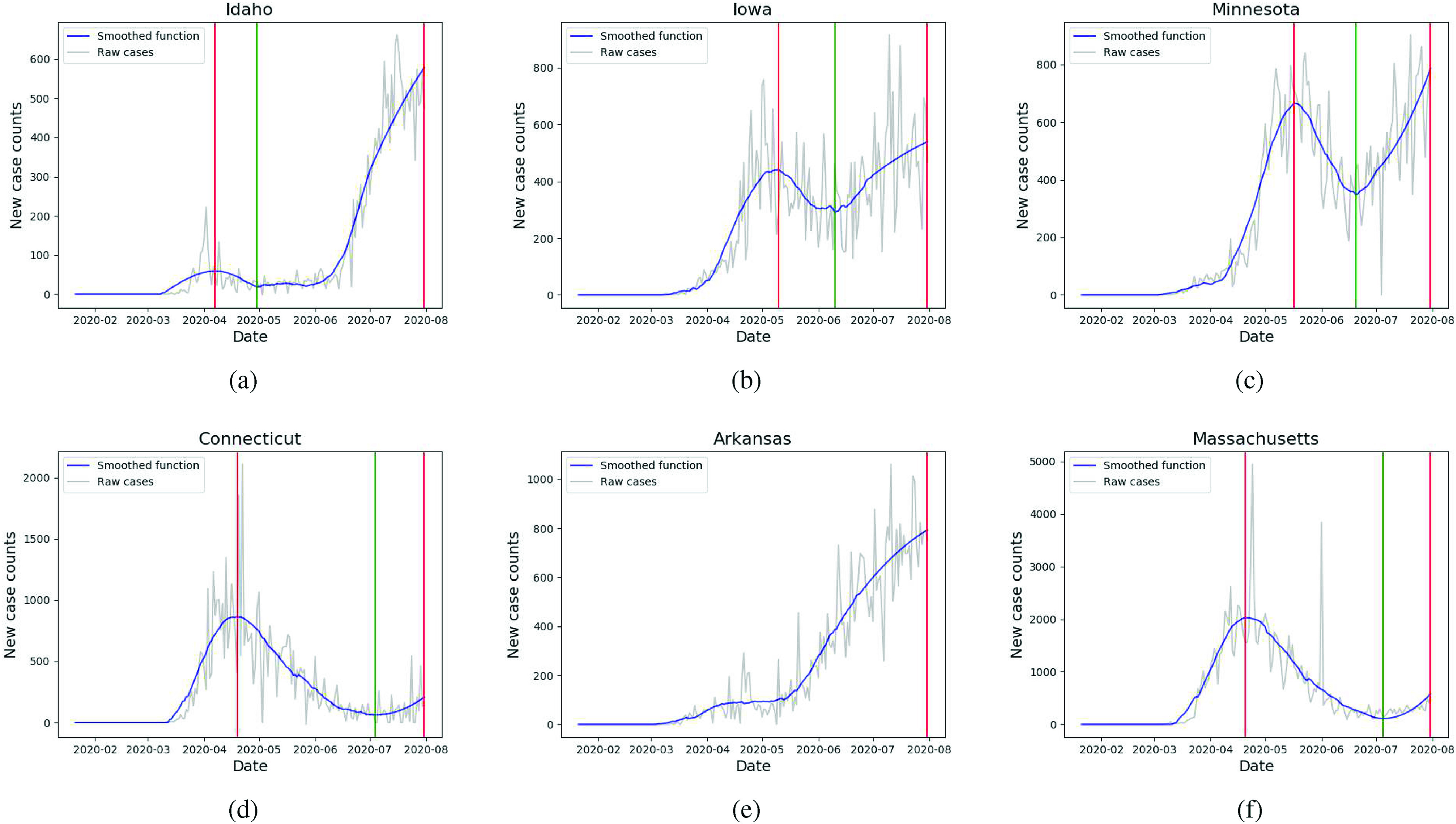
Smoothed time series and identified turning points for various states: (a) Idaho, (b) Iowa, (c) Minnesota, (d) Connecticut, (e) Arkansas, and (f) Massachusetts. Arkansas is in its first surge; Idaho, Iowa and Minnesota are experiencing second surges more severe than the first; Connecticut and Massachusetts are experiencing new increases in cases with peak ratios greater than 0.2, as defined in Sec. II. Thus, they are determined to be experiencing second surges.

In April, hierarchical clustering determines the existence of three clusters of U.S. states, displayed in Fig. 3(a). The first contains Alaska, Hawaii, Idaho, Louisiana, Montana, and Vermont, all of which have declining new case trajectories. Idaho, seen in Fig. 4(a), displays behavior typical of the cluster, experiencing a peak in early April and steadily decreasing for the remainder of the month. The second cluster consists of Iowa, Kansas, Minnesota, and Nebraska, all of which have steep increases in new case counts. Iowa and Minnesota are depicted in Figs. 4(b) and 4(c), respectively. The final cluster contains all 41 remaining states and two subclusters of high self-similarity. The first subcluster contains states whose trajectories are concave down with a local peak in April. Georgia, Pennsylvania, and Connecticut depicted in Figs. 1(b), 1(g), and 4(d), respectively, are typical of this subcluster. The second subcluster consists of states with moderately increasing trajectories, such as Mississippi and Arizona, depicted in Figs. 1(a) and 1(k), respectively.

In May, we exclude Alaska, Hawaii, and Montana from the dendrogram [Fig. 3(b)], as their smoothed trajectories mostly consist of zeroes and very low counts. Again, three clusters are observed: the first and most anomalous cluster contains only New York and New Jersey, whose trajectories are significantly decreasing, as seen in Figs. 1(i) and 1(j), respectively. The second cluster contains Arkansas [Fig. 4(e)] and Vermont; their trajectories are relatively flat at the start of May, with an uptick in the second half. All other states are contained in the final cluster, with several observable subclusters. One notable subcluster contains northeastern states Connecticut, Delaware, Massachusetts, Pennsylvania, Rhode Island, and D.C. All these states’ trajectories are steadily decreasing during May, as seen in Figs. 4(d) and 4(f) for Connecticut and Massachusetts, respectively. By contrast, another subcluster, containing North Carolina and Arizona [Figs. 1(e) and 1(k), respectively], is characterized by moderate and consistent increase in May.

In June, three clusters are again observed in Fig. 3(c). The first consists of Florida, Idaho and Montana, which display significantly increasing new cases. Figures 1(f) and 4(a) show that Florida and Idaho, respectively, experienced high growth from the beginning of June, after a prior month of moderate decrease and flat cases, respectively. In the second cluster, we again observe almost all Northeastern states, including Connecticut [4(d)], D.C., Maine [1(l)], Maryland, Massachusetts [4(f)], New Hampshire, New Jersey [1(j)], New York [1(i)], Rhode Island and Virginia. These experienced decreasing trajectories in June from an earlier peak in April. The final cluster is characterized by states with increasing trajectories. A notable subcluster that contains Mississippi, Ohio, Arkansas and others, exhibits considerable similarity in linearly increasing trajectories in June, seen in Figs. 1(a), 1(h), and 4(e), respectively.

For July [3(d)], the first of three clusters contains Arizona, Utah, Maine and Vermont. Arizona [1(k)] and Utah are experiencing decreases from their first surges, and Maine [1(l)] and Vermont from their second. The second cluster consists of Connecticut [4(d)], Hawaii and Massachusetts [4(f)], all of which exhibit growth in new cases from the beginning to end of July. The states within the final cluster almost all experienced a consistent increase in July, with more nuanced separation of trajectories occurring within the subclusters. For example, one subcluster contains California, Texas, and Florida, displayed in Figs. 4(c), 4(d), and 4(f), respectively, all of which experience a rapid increase in early July that begins to level off toward the end of July. Even New York and New Jersey experience slight increases in their cases in July, although with much lower absolute counts. Another subcluster contains Georgia, Pennsylvania and Ohio, [1(b), 1(g), 1(h), respectively], which all experience approximately linear growth in new cases. As seen in Table I, the reduced Frobenius norm for the month of July reflects less spread in the matrix as a whole, due to the large number of states with similarly increasing trajectories.

## IV. CONCLUSION

In this paper, we propose a new method for analyzing turning points and trajectories among a collection of time series. Our mathematical framework defines the characteristics of a state experiencing (or over) a second surge in COVID-19 cases. The use of semi-metrics between sets of turning points clusters states according to their differing surge behaviors and provides immediate and visible insight into the behavior of the time series collection as a whole.

This classification of behaviors is then accompanied by a close examination of the trajectories on a month-by-month basis. Here, we separate and cluster trajectories according to the relative rates of increase or decrease of new case counts. Our methodology is flexible: different smoothing techniques, metrics between data, (semi-)metrics between sets, parameters in the algorithmic framework, and clustering methods can be used to study collections of time series and identify differing surge behavior in greater generality than this application. We demonstrate this with a brief application to the Brazilian federative units in Appendix A.

Clustering the states’ trajectories on a month-by-month basis reveals consistent similarity in the cluster structure: there are always three clusters, that is, one majority cluster and two smaller clusters. The stable cluster structure over time allows one to easily observe changes in the cluster membership of individual states and determine the time frame under which new case counts in different states changed direction. For example, in May, New York and New Jersey move into a separate cluster characterized by sharply falling numbers of new cases as they introduced mask mandates.^29^

Our analysis provides insights into the evolution of COVID-19 in the United States. While previous papers have studied counts of different countries over shorter time windows,^10,11^ this paper studies the U.S. on a state-by-state basis over seven months. Within our framework, we determine that 31 states plus D.C. are experiencing second surges, of which 21 are more severe than the first surge. Thirteen states, including four of the 10 largest, are still in their first surge, with new case counts that have never materially decreased. Only two states are completely over and two partially over their first surge with no second surge as of yet. Just two states are over their second. As of the end of July, all other state counts are increasing and 32 exhibited their greatest case counts (after smoothing) on the final day of analysis. All these features are visible in Fig. 2, where five (sub)clusters correspond to these five possible surge behaviors.

The similarities in Fig. 2 can help identify common characteristics of the states that have most and least successfully managed COVID-19. New York and New Jersey, like many of the Northeastern states, experienced peaks in new COVID-19 cases in early April. Unlike other Northeastern states, these two reduced their new cases substantially and have avoided a second surge. Massachusetts and Delaware have experienced small second surges in July, 28.5% and 55.7% of their first peak, respectively.

By contrast, California, Texas, Florida, and Georgia are the four states that managed the growth of new COVID-19 cases poorly: their case counts are the highest in the nation. California and Texas limited restrictions despite cases that never stopped increasing and then reinstated them amid record counts.^30–32^ With cases *per capita* greater than California and Texas, Georgia remains in its first surge, having overturned local mask mandates in July.^33^ After an early first surge, Florida reduced restrictions and has since experienced a long and steep second surge, including the highest single day counts of any state.^34^ Figures 3(c) and 3(d) place these states in poorly performing clusters, while Fig. 2 shows the long second surge of Florida and the continuous first surges of California, Texas, and Georgia.

Overall, this paper introduces a new method for analyzing second surge behavior in a collection of time series and provides new insights into the spread of COVID-19 in the U.S. Early in 2020, many states believed that COVID-19 would resolve quickly. Few predicted that Florida’s second surge, for example, would be so much more severe than its first. Nonetheless, this is a highly infectious virus, and even countries that reported zero counts have since observed recurrences.^35^ As further surges jeopardize both citizen safety and economic recovery, individual states must closely observe the trajectory of their cases and react swiftly to minimize the potential for increasing case counts. We predict that many state governments will learn their lesson from the second surge and be cautious in observing new case counts. Vigilance going forward is necessary, and we hope states learn this in their response.

## Figures and Tables

**FIG. 5.**
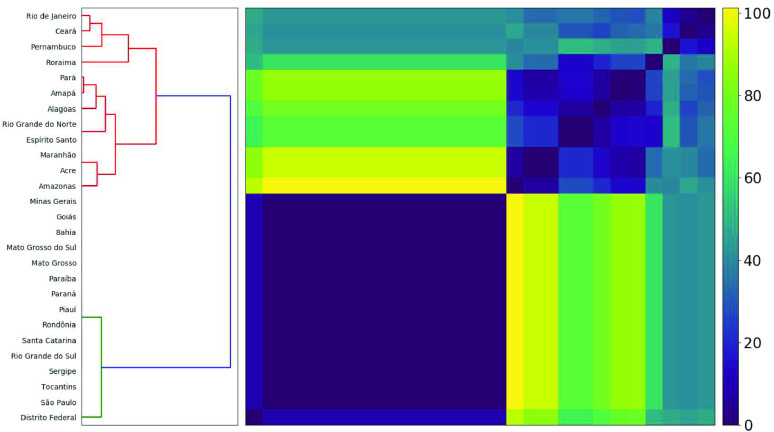
*Surge behavior matrix*, defined in Sec. II, applied to the 27 federative units of Brazil. Three clusters of time series are identified with the following behaviors: 14 states plus the federal district in their first surge, nine states coming down from their first surge, three states that are beyond their first surge and are now experiencing a second surge.

## Data Availability

The data that support the findings of this study are openly available in Refs. 36 and 37.

## APPENDIX A: COVID-19 IN BRAZIL

In this brief section, we demonstrate the generality of our method by applying it to the 27 federative units of Brazil. Our data span from 02/25/2020 to 07/31/2020, a period of 158 days. In Fig. 5, we implement hierarchical clustering on the 27×27 turning point matrix $D^{TP}$ defined in Sec. II. In contrast with the U.S., we determine that a majority of 14 states plus the federal district are in their first surge, with new case counts that have never materially decreased. Just three states are in their second surge—Ceará, Pernambuco, and Rio de Janeiro, while nine states are decreasing from their first surge. Unlike U.S. states such as Florida, the second surges are moderate and are of comparable severity to their first surges. Like the U.S., we notice significant similarity based on geography. Of the nine states

**Algorithm 1:** Turning point identification (step 1)

Given: a time series x(t)∈R
Form a smoothed time series: $\hat{x}(t)$ = Savitzky--Golay(x(t));
Data preprocessing: **if** $\hat{x}(t)<0,then\hat{x}(t)=0$;
Initialize: state =, Current TP = 1, PeakSet = empty,
TroughSet = {1};
**While** Current TP <T **do**
Set $t_{0}$ = Current TP; Flag = false;
**for** $t_{1}=t_{0}+1$ to T **do**
**if** state = TroughState **and** $t_{1}$ satisfies (1) **and** $\hat{x}(t_{1})>\hat{x}(t_{0})$ **then**
state = PeakState;
Append $t_{1}$ to PeakSet;
Current TP = $t_{1}$; Flag = true;
**Break for**
**else if** state = TroughState **and** $t_{1}$ satisfies (2) **and** $\hat{x}(t_{1})<\hat{x}(t_{0})$
**then**
Append $t_{1}$ to TroughSet;
Remove $t_{0}$ from TroughSet;
Current TP = $t_{1}$; Flag = true;
**Break for**
**else if** state = PeakState **and** $t_{1}$ satisfies (2) **and** $\hat{x}(t_{1})<\hat{x}(t_{0})$ **then**
state = TroughState;
Append $t_{1}$ to TroughSet;
Current TP = $t_{1}$; Flag = true;
**Break for**
**else if** state = PeakState **and** $t_{1}$ satisfies (1) **and** $\hat{x}(t_{1})>\hat{x}(t_{0})$ **then**
Append $t_{1}$ to PeakSet;
Remove $t_{0}$ from PeakSet;
Current TP = $t_{1}$; Flag = true;
**Break for**
**if** Flag = false **then**
**Break while**
Output PeakSet and TroughSet.

**Algorithm 2:** Turning point refinement (step 2)

TPSet = Sort(PeakSet ∪ TroughSet); ▹ Indexing begins
from 1
Initialize: CurrentPeakIndex = 2; ▹ Begin the peak ratio
refinement
**While** CurrentPeakIndex ≤ Length(TPSet) - 2 **do**
i = CurrentPeakIndex, $t_{1}$ = TPSet(i), $t_{3}$ = TPSet(i+2);
**if** $\frac{\hat{x}(t_{3})}{\hat{x}(t_{1})}\geq \delta$ **then**
CurrentPeakIndex = i+2;
**else if** $\frac{\hat{x}(t_{3})}{\hat{x}(t_{1})}<\delta$ **and** i+2 = Length(TPSet) **then**
Remove $t_{3}$ from PeakSet;
TPSet=Sort(PeakSet ∪ TroughSet);
**else if** $\frac{\hat{x}(t_{3})}{\hat{x}(t_{1})}<\delta$ **and** i+2 Length(TPSet) **then**
$t_{2}$ = TPSet(i+1), $t_{4}$ = TPSet(i+3);
**if** $\hat{x}(t_{2})\leq \hat{x}(t_{4})$ **then**
Remove $t_{4}$ from TroughSet;
**else**
Remove $t_{2}$ from TroughSet;
Remove $t_{3}$ from PeakSet;
TPSet = Sort(PeakSet ∪ TroughSet);
Initialize: CurrentIndex = 1; ▹ Begin the log-grad refinement
**while** CurrentIndex < Length(TPSet) **do**
i = CurrentIndex, $t_{0}$ = TPSet(i), $t_{1}$ = TPSet(i+1);
**if** $|\log-\mathrm{grad}(t_{0},t_{1})|<\epsilon$ **then** ▹ See Equation (3)
Remove $t_{0}$ and $t_{1}$ from both TroughSet and PeakSet;
TPSet = Sort(PeakSet ∪ TroughSet);
**else**
CurrentIndex = i+1;
Output PeakSet and TroughSet.

that are decreasing at the end of the data period, six are in the north region: Acre, Amapá, Amazonas, Pará, Rondônia, and Roraima. On the final day of analysis, 15 states exhibited their greatest new case counts (after smoothing).

## APPENDIX B: ALGORITHMIC DESCRIPTION OF METHODOLOGY

In this section, we provide an algorithmic presentation of the computational steps taken for the determination of second surge behavior, described in Sec. II. Algorithm 1 describes the first step of Sec. II A, where an alternating sequence of peaks and troughs is determined. Algorithm 2 describes the second step, where this list is refined. In our implementation, we choose three parameters l=17,δ=0.2,ϵ=0.01. Equations (1) and (2) define necessary conditions in the first step, while (3) defines a necessary condition in the second step.

## References

[1] See https://ourworldindata.org/coronavirus-source-data for “Our World in Data” (2020) (accessed August 1, 2020).

[2] R. L. Haffajee and M. M. Mello, “Thinking globally, acting locally—The U.S. response to Covid-19,” N. Engl. J. Med. 382, e75 (2020). 10.1056/NEJMp2006740 32240580

[3] M. Iati *et al.*, “All 50 U.S. states have taken steps toward reopening in time for Memorial Day weekend,” The Washington Post (2020), see https://www.washingtonpost.com/nation/2020/05/19/coronavirus-update-us (May 20, 2020).

[4] R. Meyer and A. C. Madrigal, “A devastating new stage of the pandemic,” The Atlantic (2020), see https://www.theatlantic.com/science/archive/2020/06/second-coronavirus-surge-here/613522 (June 25, 2020).

[5] L. L. Maragakis, “First and second waves of coronavirus,” Johns Hopkins Medicine (2020), see https://www.hopkinsmedicine.org/health/conditions-and-diseases/coronavirus/first-and-second-waves-of-coronavirus (June 24, 2020).

[6] R. M. da Silva, C. F. de Oliveira Mendes, and C. Manchein, “Scrutinizing the heterogeneous spreading of COVID-19 outbreak in Brazilian territory,” medRxiv (2020). 10.1088/1478-3975/abd0dc 33276353

[7] I. Bharali *et al.*, “India’s policy response to COVID-19,” The Center for Policy Impact in Global Health (2020), see http://centerforpolicyimpact.org/our-work/the-4ds/indias-policy-response-to-covid-19 (June 2020).

[8] H. W. Hethcote, “The mathematics of infectious diseases,” SIAM Rev. 42, 599–653 (2000). 10.1137/S0036144500371907

[9] G. Chowell, L. Sattenspiel, S. Bansal, and C. Viboud, “Mathematical models to characterize early epidemic growth: A review,” Phys. Life Rev. 18, 66–97 (2016). 10.1016/j.plrev.2016.07.005 27451336 PMC5348083

[10] C. Manchein, E. L. Brugnago, R. M. da Silva, C. F. O. Mendes, and M. W. Beims, “Strong correlations between power-law growth of COVID-19 in four continents and the inefficiency of soft quarantine strategies,” Chaos 30, 041102 (2020). 10.1063/5.0009454 32357675 PMC7192349

[11] J. A. T. Machado and A. M. Lopes, “Rare and extreme events: The case of COVID-19 pandemic,” Nonlinear Dyn. 100, 2953–2972 (2020). 10.1007/s11071-020-05680-w PMC7229440 32427206

[12] N. James and M. Menzies, “Cluster-based dual evolution for multivariate time series: Analyzing COVID-19,” Chaos 30, 061108 (2020). 10.1063/5.0013156 32611104 PMC7328914

[13] A. Vazquez, “Polynomial growth in branching processes with diverging reproductive number,” Phys. Rev. Lett. 96, 038702 (2006). 10.1103/PhysRevLett.96.038702 16486783

[14] R. Moeckel and B. Murray, “Measuring the distance between time series,” Physica D 102, 187–194 (1997). 10.1016/S0167-2789(96)00154-6

[15] G. J. Székely, M. L. Rizzo, and N. K. Bakirov, “Measuring and testing dependence by correlation of distances,” Ann. Stat. 35, 2769–2794 (2007). 10.1214/009053607000000505

[16] C. F. Mendes and M. W. Beims, “Distance correlation detecting Lyapunov instabilities, noise-induced escape times and mixing,” Physica A 512, 721–730 (2018). 10.1016/j.physa.2018.08.028

[17] C. F. O. Mendes, R. M. da Silva, and M. W. Beims, “Decay of the distance autocorrelation and Lyapunov exponents,” Phys. Rev. E 99, 062206 (2019). 10.1103/PhysRevE.99.062206 31330581

[18] K. Shang, B. Yang, J. M. Moore, Q. Ji, and M. Small, “Growing networks with communities: A distributive link model,” Chaos 30, 041101 (2020). 10.1063/5.0007422 32357655 PMC7192348

[19] J. H. Ward, “Hierarchical grouping to optimize an objective function,” J. Am. Stat. Assoc. 58, 236–244 (1963). 10.1080/01621459.1963.10500845

[20] G. J. Székely and M. L. Rizzo, “Hierarchical clustering via joint between-within distances: Extending Ward’s minimum variance method,” J. Classif. 22, 151–183 (2005). 10.1007/s00357-005-0012-9

[21] A.-M. Madore *et al.*, “Contribution of hierarchical clustering techniques to the modeling of the geographic distribution of genetic polymorphisms associated with chronic inflammatory diseases in the Québec population,” Public Health Genomics 10, 218–226 (2007). 10.1159/000106560 17895627

[22] M. Kretzschmar and R. T. Mikolajczyk, “Contact profiles in eight European countries and implications for modelling the spread of airborne infectious diseases,” PLoS One 4, e5931 (2009). 10.1371/journal.pone.0005931 19536278 PMC2691957

[23] H. Alashwal, M. E. Halaby, J. J. Crouse, A. Abdalla, and A. A. Moustafa, “The application of unsupervised clustering methods to Alzheimer’s disease,” Front. Comput. Neurosci. 13, 31 (2019). 10.3389/fncom.2019.00031 31178711 PMC6543980

[24] H. Muradi, A. Bustamam, and D. Lestari, “Application of hierarchical clustering ordered partitioning and collapsing hybrid in Ebola virus phylogenetic analysis,” in *2015 International Conference on Advanced Computer Science and Information Systems (ICACSIS)* (IEEE, 2015).

[25] R. Rizzi, P. Mahata, L. Mathieson, and P. Moscato, “Hierarchical clustering using the arithmetic-harmonic cut: Complexity and experiments,” PLoS One 5, e14067 (2010). 10.1371/journal.pone.0014067 21151943 PMC2997101

[26] A. Savitzky and M. J. E. Golay, “Smoothing and differentiation of data by simplified least squares procedures,” Anal. Chem. 36, 1627–1639 (1964). 10.1021/ac60214a047

[27] S. A. Lauer *et al.*, “The incubation period of Coronavirus disease 2019 (COVID-19) from publicly reported confirmed cases: Estimation and application,” Ann. Intern. Med. 172, 577–582 (2020). 10.7326/M20-0504 32150748 PMC7081172

[28] N. James, M. Menzies, L. Azizi, and J. Chan, “Novel semi-metrics for multivariate change point analysis and anomaly detection,” Physica D 412, 132636 (2020). 10.1016/j.physd.2020.132636 32834249 PMC7329734

[29] L. Ferré-Sadurnnd and M. Cramer, “New York orders residents to wear masks in public,” The New York Times (2020), see https://www.nytimes.com/2020/04/15/nyregion/coronavirus-face-masks-andrew-cuomo.html (April 15, 2020).

[30] J. Coleman, “California tells six additional counties to close indoor businesses, all bars,” The Hill (2020), see https://thehill.com/homenews/state-watch/506117-california-tells-six-additional-counties-to-close-indoor-businesses-all (July 6, 2020).

[31] P. Svitek, “Gov. Greg Abbott orders Texans in most counties to wear masks in public,” The Texas Tribune (2020), see https://www.texastribune.org/2020/07/02/texas-mask-order-greg-abbott-coronavirus (July 2, 2020).

[32] C. Silva, “U.S. COVID-19 deaths near 130,000; Florida and Texas report record case numbers,” NPR (2020), see https://www.npr.org/sections/coronavirus-live-updates/2020/07/05/88744 5479/u-s-covid-19-deaths-near-130-000-florida-and-texas-report-record-case-numbers (July 5, 2020).

[33] S. Neuman, “Georgia’s governor issues order rescinding local mask mandates,” NPR (2020) https://www.npr.org/sections/coronavirus-live-updates/2020/07/16/89171 8516/georgias-governor-issues-order-rescinding-local-mask-mandates (July 16, 2020).

[34] D. Hawkins, F. Somnez, L. Meckler, and M. Iati, “Florida shatters single-day infection record with 15 300 new cases,” The Washington Post (2020), see https://www.washingtonpost.com/nation/2020/07/12/coronavirus-update-us (July 13, 2020).

[35] “New Zealand has 69 active Covid cases after 13 more diagnosed,” The Guardian (2020), see https://www.theguardian.com/world/2020/aug/16/new-zealand-has-69-activ e-covid-cases-after-13-more-diagnosed (August 16, 2020).

[36] “Coronavirus (Covid-19) data in the United States,” The New York Times (2020), see https://github.com/nytimes/covid-19-data (accessed August 15, 2020).

[37] “Painel coronavírus,” Ministério da Saúde (2020), see https://covid.saude.gov.br (accessed August 25, 2020).

